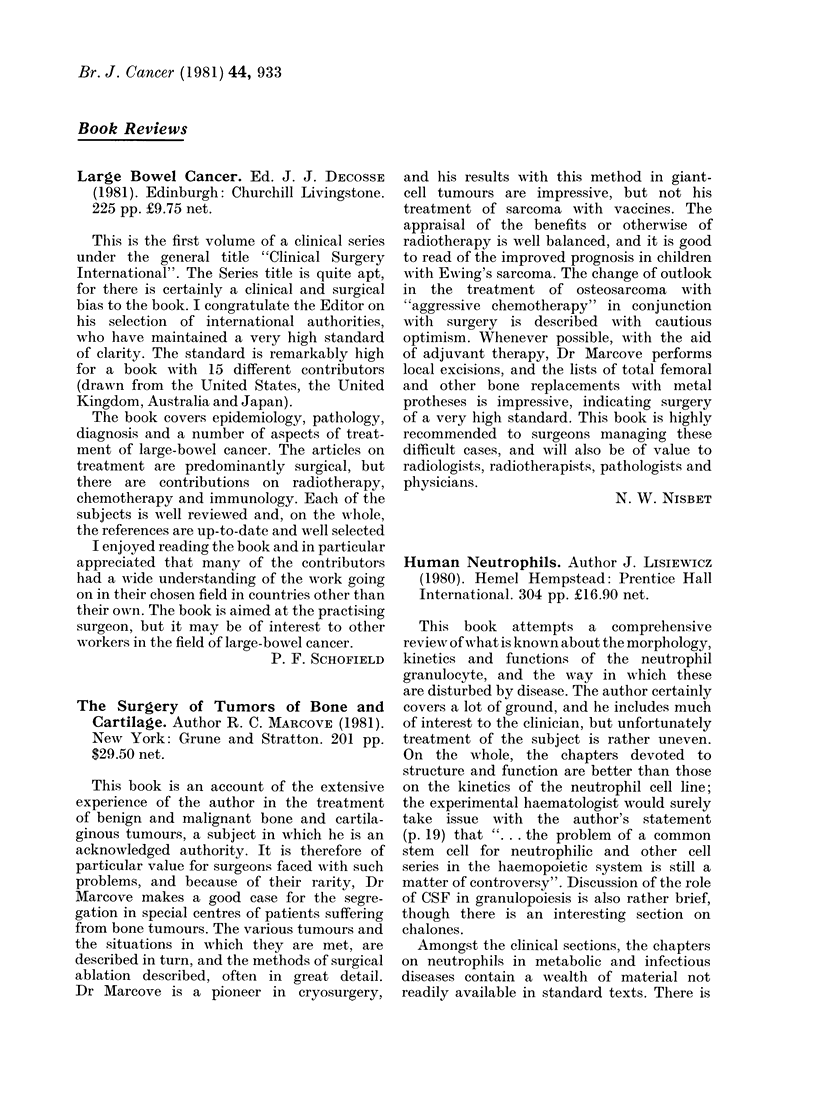# The Surgery of Tumors of Bone and Cartilage

**Published:** 1981-12

**Authors:** N. W. Nisbet


**The Surgery of Tumors of Bone and Cartilage.** Author R. C. MARCOVE (1981). New York: Grune and Stratton. 201 pp. $29.50 net.

This book is an account of the extensive experience of the author in the treatment of benign and malignant bone and cartilaginous tumours, a subject in which he is an acknowledged authority. It is therefore of particular value for surgeons faced with such problems, and because of their rarity, Dr Marcove makes a good case for the segregation in special centres of patients suffering from bone tumours. The various tumours and the situations in which they are met, are described in turn, and the methods of surgical ablation described, often in great detail. Dr Marcove is a pioneer in cryosurgery, and his results with this method in giant-cell tumours are impressive, but not his treatment of sarcoma with vaccines. The appraisal of the benefits or otherwise of radiotherapy is well balanced, and it is good to read of the improved prognosis in children with Ewing's sarcoma. The change of outlook in the treatment of osteosarcoma with "aggressive chemotherapy" in conjunction with surgery is described with cautious optimism. Whenever possible, with the aid of adjuvant therapy, Dr Marcove performs local excisions, and the lists of total femoral and other bone replacements with metal protheses is impressive, indicating surgery of a very high standard. This book is highly recommended to surgeons managing these difficult cases, and will also be of value to radiologists, radiotherapists, pathologists and physicians.

N. W. NISBET